# Research Progress on LDH Corrosion-Resistant Films on Magnesium Alloy: A Review

**DOI:** 10.3390/ma18225249

**Published:** 2025-11-20

**Authors:** Huan Li, Xue Bai, Wenjin Chen

**Affiliations:** Northwest Institute for Non-Ferrous Metal Research, Xi’an 710016, China; baixue@c-nin.com (X.B.); chenwenjin126@163.com (W.C.)

**Keywords:** magnesium alloy, LDH films, corrosion resistance, functional coating

## Abstract

As the lightest structural materials among practical metals, magnesium (Mg) alloys have broad application prospects in various fields, including automobiles, electronics, communications, aerospace and biomaterials. However, the main problem currently limiting their industrial application is poor corrosion resistance. Therefore, improving the corrosion resistance of Mg alloys has important practical value and significance. As a type of two-dimensional nanomaterial, layered double hydroxide (LDH) can serve as a micro/nanocarrier for corrosion inhibitors. Through applying LDH to constructing an in situ intelligent protective film on the surface of Mg alloy, the poor corrosion resistance of Mg alloy surfaces can be effectively improved. This paper aims to introduce the structure and properties of LDH films and provide a detailed analysis of the preparation methods and characteristics of LDH films on Mg alloy. Based on summarizing the research progress in the functional modification of LDH films for self-healing, superhydrophobic, slippery liquid-infused porous surfaces (SLIPSs) and wear-resistant coatings, the future development directions and existing challenges are discussed.

## 1. Introduction

Magnesium (Mg) alloys are some of the most important lightweight structural materials, which have so many advantages, such as a high strength-to-weight ratio, ease of machining, good biocompatibility, electromagnetic shielding properties and high recyclability [1,2,3,4]. Because of these advantages, they are widely used in the automotive, electronics, communications, aerospace and biomaterial fields [5].

However, due to their active chemical and electrochemical properties, Mg alloys are highly susceptible to oxidation and corrosion in humid and chemical environments. Moreover, the formed oxide films are porous and uneven and thus fail to provide long-term and effective protection to the Mg alloy substrate. As a result, these severely shorten the service life of Mg alloy components and limit their broader application [6,7,8,9,10]. Therefore, enhancing the corrosion resistance of Mg alloys is crucial.

In order to improve the corrosion resistance of Mg alloys, there are four main approaches at present: first, high purification, which reduces the content of harmful elements and impurities during smelting, thereby decreasing the number of susceptible phases [11]; second, alloying, which involves adding specific elements during smelting to alter the alloy composition, which improves the microstructure and slows down the corrosion rate of Mg alloys [12]; third, the addition of rare earth elements, which optimizes the microstructure and promotes the formation of dense and corrosion-resistant films [13]; and, finally, surface treatment, which involves preparing protective film layers on the Mg alloy to prevent direct contact between the substrate and corrosive media, thus achieving corrosion protection [14]. Due to its high efficiency in corrosion prevention, simple operation and low cost, surface treatment technology is widely used among these four methods. It mainly includes chemical conversion coatings [15], micro-arc oxidation coatings [16], anodizing coatings [17], electroplating coatings [18], chemical plating coatings [19], sol–gel coatings [20] and organic coatings [21]. Owing to their low cost and ease of operation, chemical conversion coatings are widely applied in the field of corrosion-resistant coatings for Mg alloys [22,23,24,25,26,27].

With progress in industry and the deepening of research, it has become increasingly challenging to meet practical application requirements solely through passive coating of corrosion-resistant layers onto Mg alloys. Therefore, it is of great significance to prepare intelligent protective coatings that can be loaded with corrosion inhibitors to achieve active protection. As a type of chemical conversion coating, layered double hydroxide (LDH) leverages its unique ion loading and ion exchange capabilities to actively release corrosion inhibitor ions and capture corrosive ions, thereby significantly enhancing the corrosion resistance of the substrates. Accordingly, this feature is a research hotspot in intelligent anti-corrosion coatings for Mg alloys [28,29,30]. This review systematically summarizes the preparation methods for LDH films on Mg alloys, focuses on the functionalization of LDH films and proposes further research directions for LDH films in the field of corrosion protection for Mg alloys.

## 2. Structure and Performance Characteristics of LDH

**Figure 1:**
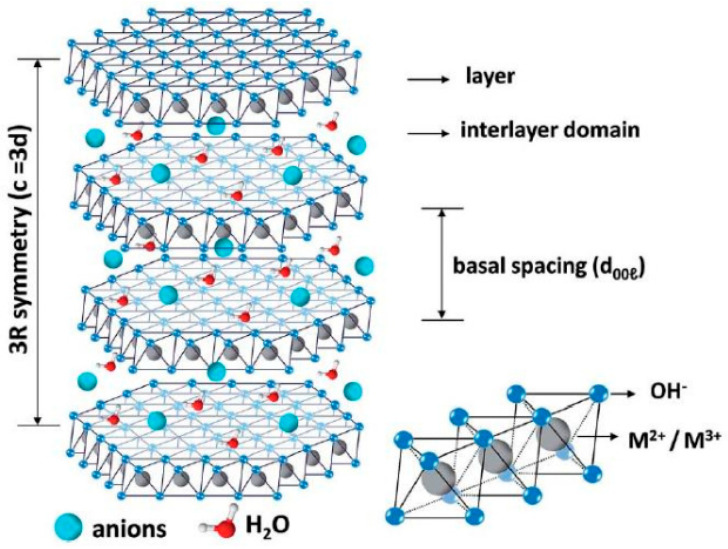
A schematic diagram of the structure of LDHs [32].

LDH, also known as hydrotalcite, is a two-dimensional layered nanomaterial with the characteristics of small size, high capacity and ease of modification. As shown in Figure 1, it consists of metal hydroxide layers interspersed with interlayer anions and water molecules. A typical layered structure is represented by the molecular formula [M^2+^_1-x_M^3+^_x_(OH)_2_]^x+^(A^n−^)_x/n_·mH_2_O, where M^2+^ represents divalent metal cations (e.g., Mg^2+^, Zn^2+^, Fe^2+^, Co^2+^, Cu^2+^), M^3+^ represents trivalent metal cations (e.g., Al^3+^, Cr^3+^, Ti^3+^, Ni^3+^, Fe^3+^, Ga^3+^) and A^n−^ represents interlayer anions (either inorganic or organic), such as CO_3_^2−^, NO_3_^−^, Cl^−^, OH^−^, SO_4_^2−^, PO_4_^3−^, C_6_H_4_(COO)_2_^2−^ and complex ions. The ratio *x*/*n* denotes the molar ratio of M^3+^/(M^2+^ + M^3+^) and is determined by the proportion of the two metal cations, while *m* represents the molar amount of interlayer water molecules. Due to the partial substitution of M^3+^ for M^2+^, the hydroxide layers acquire a positive charge, and the intercalation of anions ensures overall charge neutrality [31,32].

Due to its unique layered molecular structure, LDH possesses numerous excellent properties: (1) Loadability. Layered LDH can serve as an excellent nanocarrier and can be functionalized further through loading with corrosion inhibitors. (2) Adjustable layer composition. The divalent and trivalent metal cations in the main layers of LDH are diverse and interchangeable, which enables the adjustment of the metal cation types in the layers according to specific needs, such as Mg-Al LDHs [33], Zn-Al LDHs [34] and Li-Al LDHs [35]. (3) Exchangeable interlayer anions. The affinity of different interlayer anions for the layer plates varies significantly. Meanwhile, interlayer anions with lower affinity can be replaced by other anions. As studies show, the order of interlayer anion affinity in LDH is CO_3_^2−^ > SO_4_^2−^ > HPO_4_^2−^ > OH^−^ > F^−^ > Cl^−^ > Br^−^ > NO_3_^−^, indicating that CO_3_^2−^ binds most tightly to the layer plates of LDH, while NO_3_^−^ is the easiest to replace with other ions [36,37,38].

**Figure 2:**
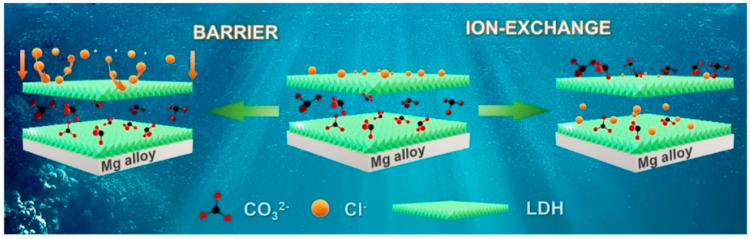
Barrier and ion exchange in magnesium alloy LDH films in corrosive environment [9].

Thus, LDH not only enables the loading and release of corrosion inhibitors but also actively captures corrosive anions from the external environment, thereby preventing damage to the substrate caused by these anions. As shown in Figure 2, the corrosion resistance of the Mg alloy matrix is enhanced through two primary mechanisms: (1) the barrier effect, which prevents the contact between corrosive ions and the matrix, and (2) the ion exchange effect, which captures corrosive anions and promotes their interlayer exchange [9].

## 3. Preparation Method for LDH Films on Mg Alloys

Currently, the common preparation methods for preparing LDH films on Mg alloys include the hydrothermal method [26,39,40,41,42,43,44,45], the steam method [46,47,48,49,50,51,52], the impregnation method [53,54,55,56], the electrochemical deposition method [26,57,58,59] and the co-precipitation method [60,61]. Among these methods, the co-precipitation method requires the pre-synthesis of LDH slurries, whereas the other methods enable the in situ formation of LDH films on the Mg alloy. Overall, each preparation method has its unique process and applicable scenarios.

### 3.1. Hydrothermal Method

Hydrothermal synthesis is a method that involves placing Mg alloy substrates and precursor solutions together in a sealed hydrothermal reactor. Under high-temperature and high-pressure conditions, LDH films form in situ on the substrate. Typically, the Mg alloy substrate provides the Mg source, while the precursor solution supplies a diverse range of metal cations and base sources. Owing to the flexibility of the precursor solution composition, the hydrothermal method is not only suitable for preparing LDH films on various magnesium alloys but also enables the one-step preparation of anionic intercalated LDH films. This versatility makes it the most widely used method for the in situ growth of LDH films [26]. In recent years, researchers have employed the hydrothermal method to prepare LDH films with different metal cations and interlayer anions on Mg alloys. Furthermore, the corrosion resistance properties have been investigated as well.

**Figure 3:**
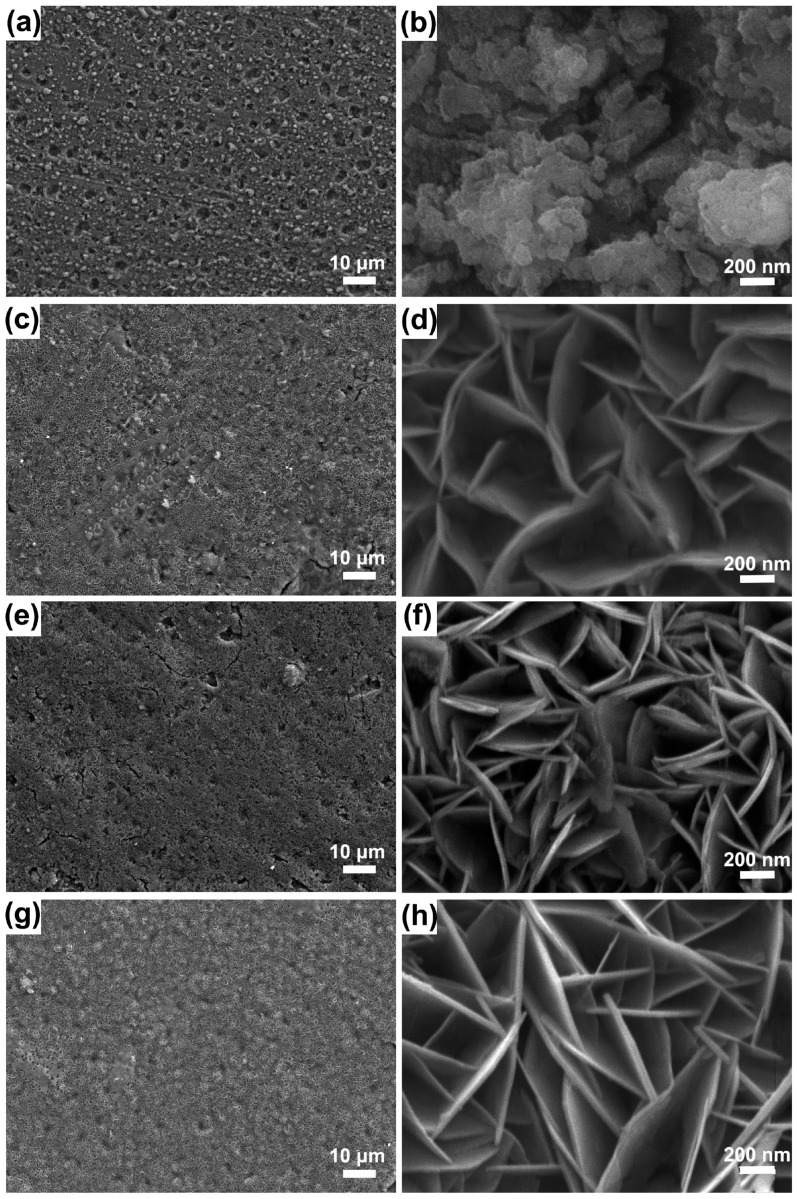
SEM surface morphology images of the anodized AZ31 magnesium alloy and Mg-M LDHs at low and high magnification: (**a**,**b**) anodized magnesium alloy; (**c**,**d**) Mg-Fe LDHs; (**e**,**f**) Mg-Cr LDHs; (**g**,**h**) Mg-Al LDHs [39].

Pan et al. [39] fabricated three types of highly oriented layered double hydroxide (LDH) films (Mg-Al, Mg-Cr and Mg-Fe) on the surface of anodized AZ31 magnesium alloy. Figure 3 presents scanning electron microscopy (SEM) images of these LDH films at low and high magnification. From the high-magnification images, it can be clearly observed that all LDH films exhibit a unique “petal-like” morphology. Specifically, the “lamellae” of Mg-Al LDH are relatively flat, while those of Mg-Fe LDH and Mg-Cr LDH are more curled. Comparison of the low-magnification images reveals that the formation of the LDH layer effectively seals the porous structure of the anodic oxide film, among which the Mg-Al LDH film exhibits the best morphological compactness.

**Figure 4:**
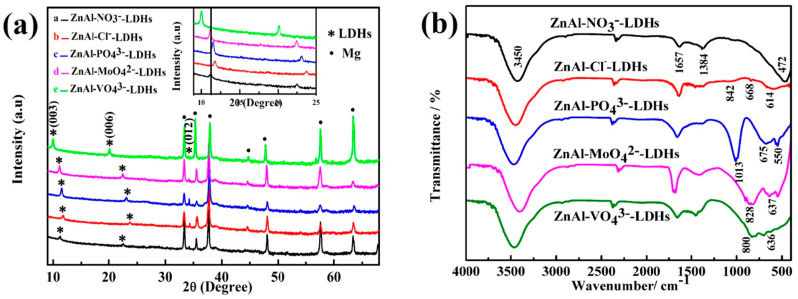
(**a**) XRD patterns and (**b**) FT-IR spectra of the ZnAl-LDHs films intercalated with different anions [40].

**Figure 5:**
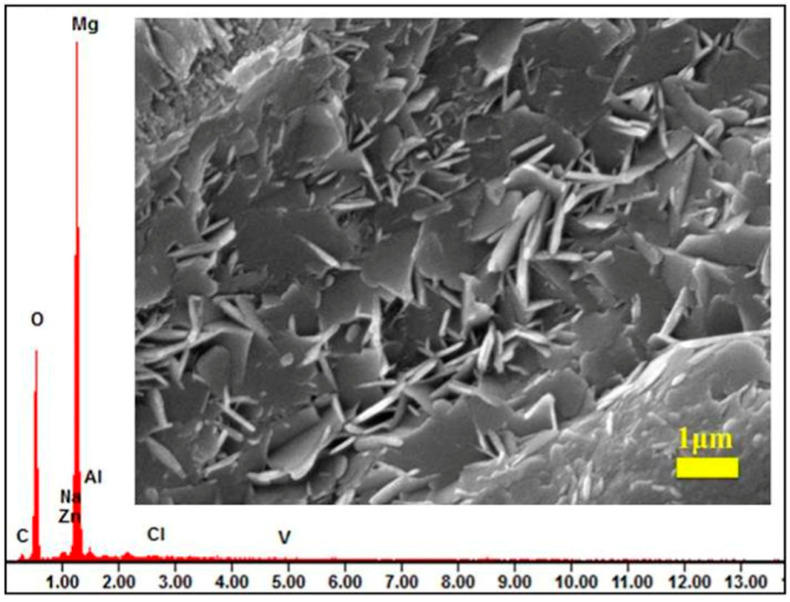
SEM images and the corresponding EDS spectra of Zn-Al LDHs intercalated with VO_4_^3−^ anions after corrosion [40].

In order to verify the successful fabrication of LDH multilayered structures and their functional characteristics, Tang et al. [40] synthesized five types of Zn-Al LDH films intercalated with different anions on AZ31 magnesium alloy substrates via a hydrothermal method. As shown in Figure 4a, XRD analysis revealed that all samples exhibited characteristic (003)/(006) diffraction peaks corresponding to the LDH phase [41], confirming the successful formation of a layered structure. The FT-IR spectra (Figure 4b) displayed characteristic vibration bands of intercalated anions, such as the V-O-V stretching vibration of VO_4_^3−^ at 636 cm^−1^, indicating the successful incorporation of anions into the interlayer galleries via ion exchange. Furthermore, SEM combined with EDS analysis (Figure 5) demonstrated that the ZnAl-VO_4_^3−^ LDHs sample retained an intact nanosheet morphology after corrosion, and the detection of Cl^−^ from the corrosive medium using EDS confirmed the active capture of chloride ions and the simultaneous release of inhibitor anions (VO_4_^3−^/Cl^−^ exchange) within the LDH interlayers.

Wu et al. [42] prepared Ce-doped Mg-Al LDH films in situ on an anodized AZ31 Mg alloy. Subsequently, vanadate intercalation was achieved through ion exchange reactions, thereby successfully preparing a Ce^3+^ and V_2_O_7_^4−^ dual-doped Mg-Al-Ce-V_2_O_7_^4−^ LDH film. Electrochemical test results indicated that the dual-doped film exhibited the highest corrosion potential and the lowest corrosion current density among the tested samples.

Wu et al. [43] achieved in situ synthesis of an Y (yttrium)-doped MXenes/Mg Al-LDH composite coating on the AZ31 Mg alloy. Studies have demonstrated that this composite coating not only exhibits excellent corrosion resistance but also possesses self-healing capabilities.

Zhao et al. [44] prepared Mg-Al LDH films and investigated the effects of the reaction parameters on the corrosion resistance of the LDH films through orthogonal experiments and range analysis. The results indicated that the reaction temperature had the most significant impact on the quality of the LDH films. Furthermore, suitably high temperatures, longer reaction times, higher aluminum source concentrations and higher pH values were found to be beneficial for forming high-quality LDH films.

Hydrothermal methods are capable of forming continuous, dense, highly crystalline and well-bonded LDH films on Mg alloys, thereby significantly improving their corrosion resistance. However, hydrothermal reactions typically require high-temperature and high-pressure reactors, which restrict the industrial implementation of LDH films on Mg alloys. To address this challenge, Shulha et al. [45] innovatively synthesized Mg-Al LDHs on AZ91 Mg alloy by using organic chelating agents (sodium nitrotrisacetic acid (NTA) and ethylenediaminetetraacetic acid (EDTA)), which can control the number of Mg^2+^ and Al^3+^ ions binding with hydroxyl groups in the solution. Ultimately, in the end, they successfully prepared Mg-Al LDH films at room temperature (25 °C). This study provides valuable insights for optimizing LDH preparation processes further to achieve their industrial application.

### 3.2. Steam Method

**Figure 6:**
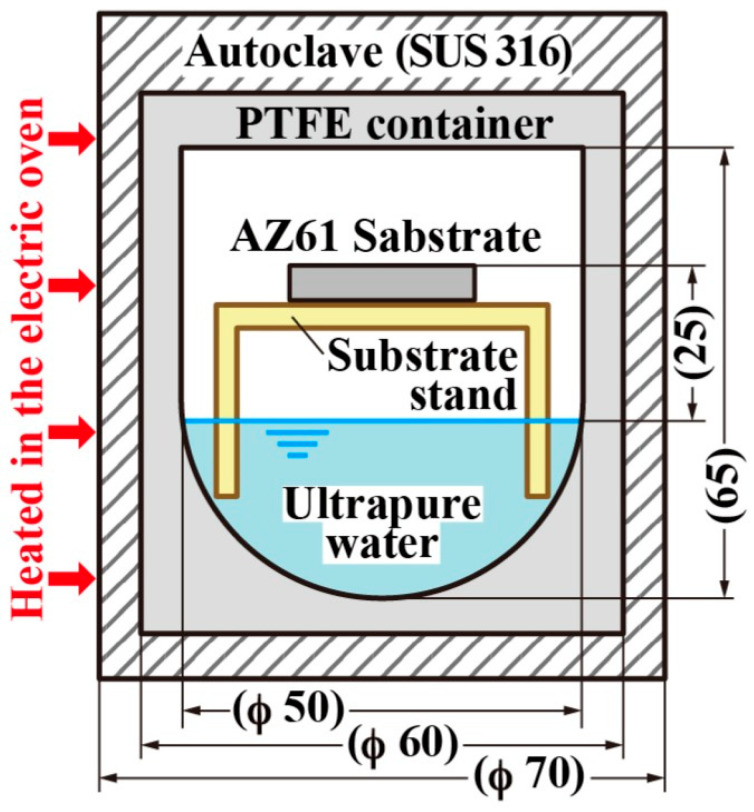
Preparation method of steam coating [50].

The steam method involves placing Mg alloys in a vapor-phase environment containing pure water or specific chemical reagents so that these chemicals react with the Mg alloys to form LDH films [46]. Notably, using this method, dense, intact, highly crystalline and well-bonded films can be formed in situ without damaging the Mg alloys. In fact, this method was first proposed by Ishizaki et al. in 2013 [47] and has been proven to form highly corrosion-resistant LDH films on Mg alloys. Since the LDH film grows directly from the Mg alloy surface, it exhibits excellent adhesion to the substrate and stability, thereby providing a reliable corrosion barrier. Furthermore, research has shown that factors such as the reaction time [48], base alloy aluminum content [49], steam pressure [50], CO_2_/CO_3_^2−^ [51] and pretreatment method [52] all affect the structure and performance of LDH films prepared through the steam method. The preparation method of steam coating is shown in Figure 6 [50].

Takahiro Ishizaki et al. [49] used the steam method to prepare LDH films on Mg alloys with different aluminum contents (AZ31, AZ61 and AZ91) and investigated the relationship between the Al content in the Mg alloys, the quantity of LDH films and corrosion resistance. Specifically, the study revealed that the film formed on AZ61 had the highest number of LDH crystallites, with a corrosion current density as low as 2.37 × 10^−8^ A/cm^2^ and a corrosion potential of −1.07 V, thus exhibiting superior corrosion resistance.

Nakamura et al. [50] investigated the effect of steam pressure variations on the structure and corrosion resistance of LDH films on the AZ61 magnesium alloy. This study revealed that steam pressure significantly influences the film structure and corrosion resistance. Specifically, corrosion resistance declines with increasing steam pressure, while an excessively low steam pressure also leads to a decrease in corrosion resistance. Therefore, adjusting the steam pressure in the reactor can control the film’s crystal growth and density to enhance the corrosion resistance of the LDH coating.

Ma et al. [51] prepared LDH films on AZ91D Mg alloy with an Al(NO_3_)_3_ solution as the steam source. The results demonstrated that increasing the Al(NO_3_)_3_ concentration could enhance the LDH content in the coating, thereby increasing the density and thickness of the film. In turn, this structural improvement effectively prevents the penetration of corrosive media, leading to a significant enhancement in the magnesium alloy’s corrosion resistance. More specifically, when the Al(NO_3_)_3_ concentration reaches 100 mmol/L, the coating exhibits the lowest corrosion current density and the best corrosion resistance.

Pan et al. [52] prepared a Mg-Al LDH film on AM50 Mg alloy using oxalic acid pretreatment combined with a steam coating method. Their study revealed that oxalic acid pretreatment significantly increased the proportion of intermetallic compounds on the alloy surface, thereby promoting LDH film nucleation and growth. Furthermore, when the acid washing time was 45 s, the film achieved the maximum thickness and the best corrosion resistance, and the corrosion current density was reduced by three orders of magnitude compared to that for the untreated substrate.

The steam method for preparing LDH films on Mg alloys can use pure water as the steam source, entailing minimal environmental pollution. However, the preparation process still requires operation in a high-pressure reactor, resulting in limited reports on its current industrial implementation. Further optimization of the steam coating preparation process and exploration of broader applications will be the focus of future research.

### 3.3. Impregnation Method

The impregnation method involves immersing the Mg alloy substrate into a reaction solution to facilitate the adsorption, deposition or reaction of solution substances with the Mg alloy to form a thin film on the substrate. Unlike the hydrothermal and steam methods, LDH films prepared through the impregnation method can be synthesized under ambient pressure. In addition, experimental parameters such as the solution pH, reaction temperature and ionic radius significantly affect the experimental outcomes.

The impregnation method, which can be classified into one-step and two-step procedures, is a widely used technique for coating preparation. Lin et al. [53] employed a one-step impregnation method to immerse AZ91 Mg alloy in a CO_2_-saturated aqueous solution, resulting in the in situ formation of Mg-Al LDH films. Specifically, electrochemical tests showed that the coated sample had a corrosion potential 0.15 V higher than that of the base Mg alloy, with the corrosion current density only one-eighth of that of the base material.

The one-step impregnation method, despite its operational simplicity, has several drawbacks, including low crystallinity, a prolonged processing time and low coating preparation efficiency. To overcome these limitations and reduce the reaction time, a two-step impregnation method has been developed. Initially, the Mg alloy is dissolved in an acidic solution to release Mg^2+^ ions, which then combine with other ions to form a precursor film.

Subsequently, the precursor film undergoes post-treatment in an alkaline solution. Finally, ion exchange is performed to form the hydrotalcite film. The two-step method is currently widely used to prepare LDH films on Mg and Mg alloys. Yu et al. [54] used a two-step method to fabricate Mg-Al LDH films on AZ91 Mg alloy. After a 72 h salt spray test, no corrosion spots were observed on the sample surface. However, this technique relies on the dissolution of the Mg alloy substrate for the Mg and Al elements in the film, thereby restricting its applicability to Al-rich Mg alloys.

To achieve in situ growth of Mg-Al LDH films on low-aluminum-content Mg alloys via an immersion method, Chen et al. [55] employed a two-step approach to fabricating highly corrosion-resistant Mg-Al LDH films on the low-Al AZ31 Mg alloy. First, pure aluminum plates were dissolved in a carbonate pretreatment solution until aluminum reached saturation to yield a pretreatment solution (pH ≈ 8). Next, NaOH solution was then added dropwise to the pretreatment solution until the pH reached 10.5, forming a post-treatment solution. Subsequently, the samples were immersed in a 60 °C pretreatment solution and continuously bubbled with CO_2_ for 30 min to form a precursor coating. Finally, the samples were reacted in an 80 °C post-treatment solution for 1.5 h to obtain a continuous and dense Mg-Al LDH film.

The LDH coatings prepared through this method exhibited excellent crystallinity, with a reaction time of approximately 2 h—half the duration of the technique reported by Yu et al. [54]. After immersion in a 0.1 M NaCl aqueous solution for 48 h, the AZ31 magnesium alloy coated with LDH showed a significantly higher corrosion potential and a lower corrosion current density than those of the uncoated alloy. These results demonstrated that the coating provides effective protection for the AZ31 Mg alloy.

Meanwhile, Chen et al. [56] investigated the growth mechanism of in situ Mg-Al LDH films prepared on the AZ31 Mg alloy via an immersion method. Research has shown that the addition of aluminum compounds to the pretreatment or post-treatment solution is critical for promoting LDH film formation.

In summary, the two-step immersion method can effectively improve the corrosion resistance of Mg alloys. However, the reaction solution contains a high concentration of CO_3_^2−^ ions, which exhibit the strongest binding affinity with LDH layers. Therefore, this poses challenges for the incorporation of corrosion inhibitors via anion exchange into the prepared LDH film. This limitation restricts the functionalization of the LDH film.

### 3.4. Electrochemical Deposition Method

Electrochemical deposition is a method for fabricating LDH films via electrochemical reactions. The resulting LDH films comprise nanowalls oriented perpendicular to the substrate. This method offers several advantages: mild reaction conditions, high phase purity, fast deposition rates, simple equipment requirements and compatibility with complex geometries [26].

Syu et al. [57] used the electrochemical deposition method to fabricate a Li-Al LDH film on AZ31 Mg alloy. The study showed that the corrosion resistance of the Li-Al LDH film correlates with its thickness; specifically, when the film thickness is ≥420 nm, the coating provides effective protection against corrosion for the substrate. However, the key materials in this method, Al-Li intermetallic compounds, are highly reactive. The preparation and storage processes for the Li^+^/Al^3+^ reaction solution are complicated.

**Figure 7:**
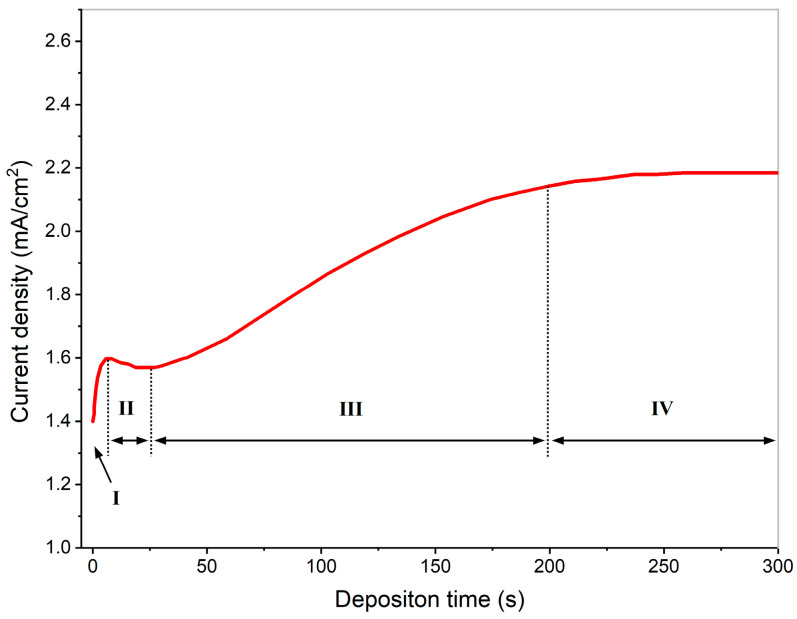
Variation in the current density with the deposition time when a potential of −1.7 V is applied in the solution of Zn^2+^/Al^3+^ [58].

Wu et al. [58] developed a simple and rapid method for the electrochemical deposition of a Zn-Al LDH film. The Zn-Al LDH film was prepared through constant-potential electrodeposition at room temperature on the AZ91D Mg alloy. The results showed that the electrodeposited film comprised Zn-Al LDH embedded into nitrate, with a thickness of 3 μm. The film exhibited a uniform and dense microstructure and excellent adhesion to the substrate. As shown in Figure 7, the film formation process was divided into four stages based on monitoring the current density changes over time: initially (I), the cathodic reduction of NO_3_^−^ or H_2_O generates OH^−^, causing a sharp increase in the current density. Subsequently (II), the deposition of Zn-Al hydroxide accompanied by H_2_ bubble formation blocks active sites, leading to a slight current density decline. The third stage (III) involves bubble desorption, which re-exposes substrate areas, while renewed reaction sites promote LDH growth and the porous interlayer structure reduces the mass transfer resistance, collectively elevating the current density slightly. Finally (IV), film thickening and diminished bubble effects stabilize the reaction system, with the current density plateau indicating completed Zn-Al LDH film formation.

Ouyang et al. [59] optimized the electrodeposition process to fabricate Mg-Al LDH films on AZ31 Mg alloy. The LDH-coated alloy showed excellent corrosion resistance in a 3.5% NaCl solution.

Electrochemical deposition is a widely used method for preparing LDH films owing to its simplicity, high efficiency and adaptability to various substrate geometries. However, it is energy-intensive and costly, while the adhesion between the film and substrate is often insufficient. Moreover, the selection of the process parameters is critical; improper choices can lead to by-products or even hinder film formation. Thus, optimizing the deposition parameters and improving film-substrate adhesion represent key research directions.

### 3.5. Co-Precipitation Method

The co-precipitation method involves first synthesizing LDH slurries from metal salts, followed by forming LDH films on Mg alloys via hydrothermal reactions. Its main advantage is precise control of the chemical composition of LDH, leading to single-crystalline LDH. Although the co-precipitation method uses hydrothermal reactions to prepare LDH films, the bonding strength between the LDH films and the substrate is weaker than that of in situ hydrothermal methods. However, the co-precipitation method can prepare LDH films on substrates where in situ hydrothermal methods cannot directly grow LDH films.

Tai et al. [60] fabricated Mg-Al-MoO_4_^2−^ LDH films on the AZ31B Mg alloy via co-precipitation and hydrothermal treatment. Their study showed that molybdate ions were successfully intercalated into the interlayers of the LDH films, serving as effective corrosion inhibitors. Without compromising the barrier properties of the LDH film, controlled release of these inhibitors was achieved, thereby slowing the corrosion of the Mg alloy.

Wang et al. [61] employed micro-arc oxidation (MAO), co-precipitation and hydrothermal treatment to fabricate a MAO-LDH composite film on AZ91 Mg alloy. They successfully synthesized MoO_4_^2−^-intercalated Mg-Al LDH slurry via co-precipitation and deposited a uniform and continuous LDH film onto the MAO film’s surface, effectively sealing its micropores. After immersion in 3.5% NaCl solution for 144 h, the MAO-LDH composite coating continued to provide effective protection for the Mg alloy.

In conclusion, the selection of a preparation method for Mg alloy LDH films should consider the material type, preparation conditions and specific application requirements. A comparison of the preparation methods, properties and application scenarios for LDH films on Mg alloys (corrosion solution: 3.5 wt% NaCl) is shown in Table 1.

## 4. Functionalization of LDH Films on Mg Alloys

LDH films usually grow perpendicular to the substrate surface in a lamellar cross-linked structure, with certain pores and gaps on the coating surface [62]. When exposed to a corrosive environment for a long time, the corrosive solution easily penetrates the coating, eventually leading to the loss of the LDH film’s protective effect on the substrate. Meanwhile, LDH films are typically thin (only a few microns thick), making them prone to detachment from the substrate under mechanical scratching. To further enhance the durability of corrosion-resistant LDH films on Mg alloys and improve the coating’s comprehensive performance, researchers have focused on endowing films with composite functions to meet various practical applications, such as self-healing, superhydrophobicity, slippery liquid-infused porous surface (SLIPS) and wear resistance properties.

### 4.1. Self-Healing LDH Films

Leveraging the unique layered structure and properties of LDH films, researchers have achieved self-healing of LDH membranes from both morphological and functional perspectives via processes such as LDH dissolution-recrystallization, anion exchange and corrosion inhibitor loading/release [63]. These methods not only enhance the film’s corrosion resistance but also extend its service life in harsh environments.

Zhu et al. [64] employed thermal diffusion surface alloying and hydrothermal treatment to integrate the intrinsic “smart” corrosion protection of LDH films with the excellent passivity of aluminum-alloyed surfaces, thereby preparing aluminum-alloyed LDH films on the AZ31 Mg alloy. This approach achieved prolonged corrosion resistance for the Mg alloy. Specifically, scratch test results revealed that upon exposure to the corrosive medium, Mg and Al dissolved to form Mg^2+^ and Al (OH)_4_^−^ ions. In turn, these ions subsequently underwent recrystallization reactions under alkaline conditions to form a reconstructed surface film in the scratched area, completing the self-healing process.

Li et al. [65] functionalized Mg-Al LDH films with the organic inhibitor N-alkyl-N-(NTA). The results showed that the corrosion potential of the NTA-treated Mg-Al LDH film was −0.437 V, significantly higher than that of the untreated coating (−0.750 V). The self-healing mechanism involves two key processes: Firstly, Mg^2+^ ions within the Mg-Al LDH react with CO_3_^2−^ to form a precipitate film that seals scratch defects. Secondly, NTA molecules adjacent to the scratches adsorb onto the Mg (0001) crystal surface and Mg oxides via both physical and chemical interactions, forming a protective barrier.

Gnedenkov et al. [66] grew LDH films in situ on the MA8 Mg alloy PEO film via the immersion method and further functionalized the LDH films with benzotriazole (BTA). The results showed that in a 3.5 wt% NaCl solution, the corrosion rate of the PEO-LDH-BTA samples was 30% lower than that of PEO samples, exhibiting the highest protective performance over a 24 h period. The study also suggested that the anti-corrosion effect and self-healing behavior of this smart composite coating might be attributed to the formation of Mg (BTA-H)_2_ complexes in defect areas first, followed by the precipitation of Mg(OH)_2_.

Song et al. [67] investigated the use of aspartic acid (ASP) and lauric acid (LA) organic acid anions as corrosion inhibitors to fabricate two types of films—ZnAl-LA LDH and ZnAl-ASP LDH—on the AZ31 Mg alloy. The results revealed that the corrosion resistance of the ZnAl-LA LDH film was markedly superior to that of the ZnAl-ASP LDH film. Compared with the Mg substrate, the corrosion current density of the ZnAl-LA LDH film decreased by two orders of magnitude. The film also exhibited a superior capacity to absorb Cl^−^ and release interlayer anions, thereby inhibiting further corrosion and enabling functional self-healing.

Zhang et al. [68] fabricated a PEO-Ce-P LDH composite film by sealing a plasma electrolytic oxidation (PEO) film with cerium and hydrothermally modifying Mg-Al LDH with phytic acid (PA). The synergistic effect of cerium and phosphate enables the composite coating to exhibit excellent corrosion resistance and a self-healing capability.

**Figure 8:**
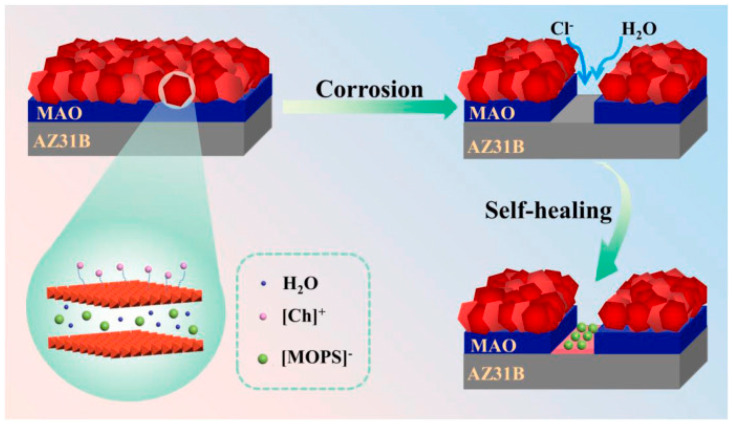
A schematic diagram of the corrosion protection mechanism of the CM-LDH/MAO composite film [69].

Liu et al. [69] first developed choline 3-morpholine propanesulfonate ([Ch][MOPS]) ionic-liquid-modified MAO/LDH composite coatings to enhance the corrosion resistance of Mg alloys, with Figure 8 illustrating the corrosion protection mechanism of the CM-LDH/MAO composite film. The results showed that the corrosion current density of the CM-LDH/MAO coating was 8.38 × 10^−10^ A/cm^2^, which was significantly lower than that of the MAO and LDH/MAO coatings. After 72 h of immersion, the scratch area was filled with dense insoluble deposits. After 120 h of immersion, the |Z| values at 0.01 Hz were even higher than those after 72 h, further confirming the coating’s self-healing effect.

Currently, although LDH self-healing films have been extensively studied, there are still several challenges. For example, they can only be loaded with small-molecule anionic corrosion inhibitors, and the loading capacity of macromolecular corrosion inhibitors is limited and relies on foreign substances. Additionally, the coatings’ long-term self-healing capability, environmental adaptability and corrosion self-warning function require further improvement.

### 4.2. Superhydrophobic LDH Films

Inspired by the ‘lotus leaf’ effect, artificial superhydrophobic surfaces can be fabricated by simultaneously constructing a micro-nano rough surface and introducing low-surface-energy materials into the substrate. Because LDH films exhibit not only a lamellar micro-nano structure but also a rough surface with abundant hydroxyl groups, they can interact with low-surface-energy substances (e.g., long-chain fatty acids and fluoroalkyl silanes) via condensation reactions to endow the coating with superhydrophobicity. This property further enhances the corrosion resistance and durability of magnesium alloy LDH films [70,71].

Wang et al. [72] grew Mg-Al LDHs loaded with different corrosion inhibitors (NO_3_^−^, VO_4_^3−^, MoO_4_^2−^) in situ on AZ31 Mg alloy and modified them further with sodium stearate (SS), lauric acid (LA) and myristic acid (MA) to fabricate superhydrophobic LDH films. The results showed that LDH films modified with low-surface-energy substances transitioned from hydrophilic (contact angle of 44.5°) to hydrophobic (water contact angle > 139°).

**Figure 9:**
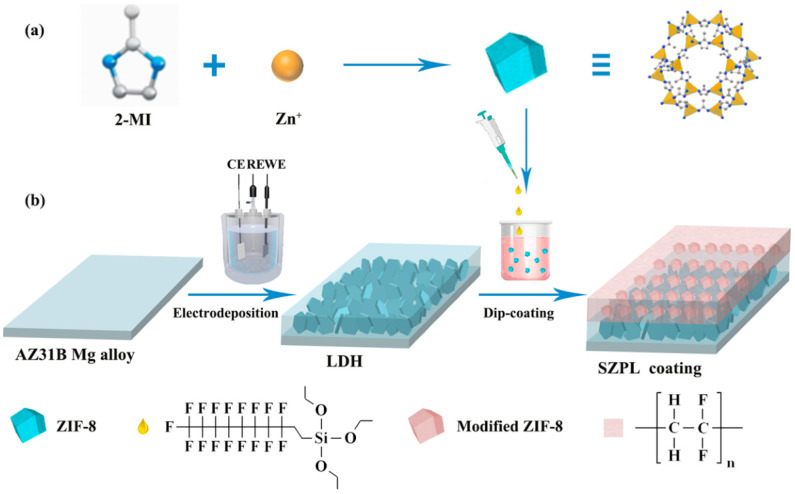
(**a**) Synthesis route of ZIF-8. (**b**) A schematic of the preparation of the SZPL coating on the Mg alloy [73].

Yin et al. [73] combined electrochemical deposition with an impregnation method to fabricate a composite coating on AZ31B Mg alloy, which consisted of a bottom LDH transition layer and a surface superhydrophobic ZIF-8 layer. The preparation procedure is depicted in Figure 9. The results revealed that the composite coating exhibited an anti-corrosion efficiency exceeding 99.9% and maintained an excellent anti-corrosion performance after immersion in a 3.5 wt% NaCl solution for 7 days.

Zhu et al. [74] embedded AZ31 Mg alloy into Zn-Al alloy powders and heated it at 673.15 K for 2 h in an argon-protected tube furnace to form a Zn-Al alloyed pretreatment film. Subsequently, Mg-Zn-Al LDH films were grown in situ via hydrothermal synthesis, and the surface was modified with lauric acid to achieve superhydrophobicity. The resulting composite LDH films integrate superhydrophobicity, anion exchange/release capability and physical barrier properties. The experimental results demonstrate excellent corrosion resistance under the combined protective effects. After modification with lauric acid, the superhydrophobic LDH-LA films exhibited the highest corrosion potential (1.227 V) and the lowest corrosion current density (0.077 mA/cm^2^).

Currently, superhydrophobic LDH coatings still suffer from a rapid decrease in the static contact angle (CA) and insufficient stability after prolonged immersion. It is necessary to select more stable functional groups to form a strong bond with LDH to reduce the surface energy.

### 4.3. Slippery Liquid-Infused Porous Surface (SLIPS) LDH Films

**Figure 10:**
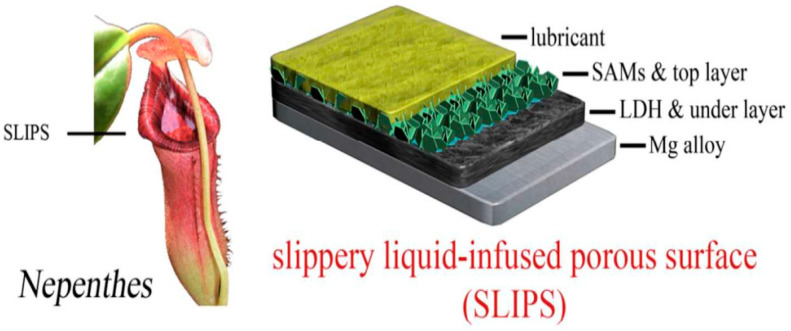
SLIPS/LDH composite coating on Mg alloy [75].

Inspired by the *Nepenthes* pitcher plant, Aizenberg et al. first proposed the concept of slippery liquid-infused porous surfaces (SLIPSs). Building on this concept, researchers fabricated a super-slippery LDH film on a magnesium alloy by infusing lubricating liquid into the micro-nano porous substrate of the LDH coating, as illustrated in Figure 10. Compared with superhydrophobic LDH coatings, the resulting film demonstrates superior high-pressure resistance and self-healing properties [75,76].

Tian et al. [77] combined in situ growth with chemical modification to fabricate a biomimetic SLIPS on a magnesium-lithium alloy (Mg-Li). The results showed that the SLIPS provided a multifunctional protective barrier to Mg-Li alloys, encompassing antifouling, self-cleaning, self-healing, impact resistance, durability and corrosion resistance, with strong adhesion to the substrate. To enhance the durability of SLIPSs on magnesium alloys and address the challenge of repairing damaged coatings, Niu et al. [78] developed a novel SLIPS coating by leveraging the self-supplementary properties of silica aerogels and LDH-based lubricants. The results reveal that the coating, after undergoing oil-paper absorption, centrifugation and grinding, can restore its lubrication performance even after repeated damage and exhibits lubricant self-replenishment capabilities. Furthermore, the SLIPS coating reduces the coverage of marine organisms and bacteria by more than 80%.

Considering that the SLIPS coating relies on the interconnected micro-cavities of the LDH surface structure for lubricant storage, Yao et al. [79] optimized the surface microstructure of Zn-Al LDH films on AZ31 magnesium alloy by adjusting the electrolyte pH value to enhance the accommodation capacity for lubricating fluids. The experimental results showed that pH value had a significant influence on the thickness and density of the LDH films. When the pH was 8, the Zn-Al LDH film had a thickness of 5.3 μm, the highest compactness and a silicone oil loading capacity of up to 0.25 mg/mm^2^. Compared with superhydrophobic surfaces (a water contact angle > 150° and a sliding angle < 10°), the SLIPS coating maintained a contact angle of approximately 110° after 14-day long-term immersion in 3.5 wt.% NaCl solution (the contact angle of the superhydrophobic surface dropped below 90°). In addition, the SLIPS coating had a corrosion current density as low as 5.01 × 10^−9^ A/cm^2^ and an impedance modulus as high as 4.92 × 10^7^ Ω·cm^2^, showing optimal corrosion resistance. This is mainly due to the stable silicone oil layer, which can continuously isolate corrosive media.

Jang et al. [80] fabricated a multilayer barrier coating system on Mg alloys, comprising a bottom PEO base layer, a middle Mg-Al LDH functional coating and a surface SLIPS coating. The composite coating exhibited superior corrosion resistance and self-healing properties.

To summarize, a key challenge with SLIPS coatings is the poor long-term stability of their lubricants: prolonged immersion causes gradual lubricant leaching. This loss impairs the coating’s protective structure, leading to obvious degradation of the protective performance, thereby limiting the coatings’ long-term service reliability in practical use.

### 4.4. Wear-Resistant LDH Films

To enhance the corrosion and wear resistance of LDH films on Mg alloys, researchers have developed a novel functional coating by integrating silica (SiO_2_) nanoparticles into SLIPS coatings.

Yao et al. [81] synthesized a sodium-benzoate-intercalated Mg-Al LDH coating on AZ31 Mg alloy via hydrothermal synthesis and subsequently modified it via superhydrophobic chemical treatment. Building on this, they fabricated SiO_2_-SLIPS-MgAl LDH composite coatings by infusing silicone oil laden with different concentrations of SiO_2_ nanoparticles into the coating matrix. The results revealed that the incorporation of SiO_2_ nanoparticles further enhanced the wear resistance of the composite coating. During the friction process, the nanoparticles, owing to their higher hardness, act as tiny rolling bearings, converting sliding friction into rolling friction and thereby significantly reducing the friction coefficient and the wear rate.

Wang et al. [82] investigated the specific impact of surface microstructure on the frictional properties by preparing LDH nanosheets coordinated with different divalent metal ions (Mg^2+^, Zn^2+^ and Co^2+^). The results indicated that the tribological properties of the LDH films were closely correlated with the coordination stability of divalent metal ions in the surface structure. Among the three LDHs, the octahedral coordination stability of the Co^2+^ ions in LDH-Co was the highest. The primary reason is that the octahedral coordination of Co^2+^ ions significantly reduces the sliding energy barrier, and their adsorption onto the probe silicon sphere is the weakest, thereby effectively reducing friction.

From the perspective of current research progress, although single functional modification (such as ion intercalation, hydrophobic modification and nanoparticle composites) can specifically improve a specific performance of LDH coatings (e.g., ion intercalation enhances corrosion inhibition, while hydrophobic modification improves environmental stability), it is difficult to meet the comprehensive requirements for “corrosion resistance–wear resistance–long-term durability” of magnesium alloys in complex service scenarios.

## 5. Conclusions and Future Perspectives

This paper provides a comprehensive overview of the advancements in the primary preparation methods for LDH films on Mg alloys and discusses the progress in the functional modification of LDH films toward self-healing, superhydrophobic, SLIPS and wear-resistant functionalities. By summarizing the comparisons and correlations between different research works, this paper aims to help researchers gain an overall understanding and provide some references in this field. Despite significant progress in research on LDH films for Mg alloys, previous investigations still have limitations, and further investigations are required in the near future.

(1) Currently, the preparation and research of LDH films remain largely confined to laboratory-scale studies. To facilitate their transition to industrial applications, further optimization of both the synthesis methods and processing procedures is imperative.
(2) Current research efforts are predominantly directed toward exploring the functional applications of LDHs, whereas systematic investigations into the intrinsic physicochemical properties of LDH films and their correlations with industrial application scenarios remain relatively limited.
(3) Despite the excellent corrosion protection of the functionalized LDH coatings under laboratory conditions, they encounter significant challenges in complex industrial scenarios. To address this, LDH coatings as a pretreatment substrate paired with a topcoat can form a composite protection system—integrating their active protection and the topcoat’s efficient passive barrier performance—meeting industrial needs for corrosion-prone metals such as magnesium alloys better and delivering a more reliable protective solution.
(4) Exploring the combination of LDH with surface treatment technologies such as plasma spraying, cold spraying and laser processing is expected to achieve the optimal performance coupling of green manufacturing, low cost, functionalization and high corrosion resistance.

## Figures and Tables

**Table 1:** Comparison of preparation methods, properties and application scenarios for LDH films on Mg alloys (corrosion solution: 3.5 wt% NaCl).

Method	Conditions	Advantages	Disadvantages	Film Quality and Core Features	Corrosion Current Density (A/cm^2^)	Functional Expandability	Application Scenarios	References
Hydrothermal treatment	High temperature, high pressure	Stable performance, high process controllability, widely used	Long reaction time, high temperature, high equipment requirements, medium cost	▪ Dense coating, strong bonding strength, good functionalization ▪ Simultaneous realization of metal doping and anion intercalation	9.12 × 10^−9^–3.026 × 10^−7^	Metal ion doping, anion intercalation	Automotive components, medical implants	[26,39,40,41,42,43,44,45]
Steam coating	High temperature, high pressure	High efficiency, environmentally friendly, low cost	High pressure, high safety requirements, limited types of Mg alloys, weak ion exchange capacity, presence of by-products	▪ Thick and dense film, uniform, good bonding, good micro-roughness ▪ In situ formation of LDH films	8 × 10^−9^–2.4 × 10^−8^	None	Mass-produced structural parts, green production scenarios	[46,47,48,49,50,51,52]
Impregnation method	Atmospheric pressure and room temperature	Simple method, easy operation, environmentally friendly, low cost	Weak functionalization, poor film durability, limited types of Mg alloys, with Al-rich grades (AZ31, AZ91)	▪ Porous film, uniformity coating, limited corrosion resistance ▪ Short-term temporary protection	2.21 × 10^−6^– 10 × 10^−6^	None	Small-batch samples, short-term storage	[26,53,54,55,56]
Electrodeposition	Atmospheric pressure and room temperature	Short reaction time, mild reaction conditions, simple equipment, coating not limited by substrate shape, suitable for large-sized components	High energy consumption, prone to by-products, high cost	▪ Uniform and dense coating, high phase purity, poor bonding ▪ Rapid and uniform film formation	2.12 × 10^−6^–7.882 × 10^−7^	Metal ion doping, anion intercalation	Components with complex shapes, large-scale production	[26,57,58,59]
Co-precipitation	High temperature, high pressure	Controllable chemical composition, wide scope of application	Complex operation, time-consuming operation, high equipment standards, high cost	▪ Poor bonding, difficult to control, uneven particle size distribution ▪ Customized precursor preparation	1.2 × 10^−7^–1.6 × 10^−7^	Metal ion doping, anion intercalation	Customized functional coatings (marine/medical applications)	[60,61]

## Data Availability

No new data were created or analyzed in this study. Data sharing is not applicable to this article.
